# Uric acid, an important antioxidant contributing to survival in termites

**DOI:** 10.1371/journal.pone.0179426

**Published:** 2017-06-13

**Authors:** Eisuke Tasaki, Hiroki Sakurai, Masaru Nitao, Kenji Matsuura, Yoshihito Iuchi

**Affiliations:** 1 Department of Applied Bioresources Chemistry, The United Graduate School of Agriculture, Tottori University, 4–101 Koyamacho-minami, Tottori, Japan; 2 Department of Biological Chemistry, Faculty of Agriculture, Yamaguchi University, 1677–1 Yoshida, Yamaguchi, Japan; 3 Department of Applied Biosciences, Graduate School of Agriculture, Kyoto University, Kitashirakawa Oiwakecho, Kyoto, Japan; 4 Graduate School of Sciences and Technology for Innovation, Yamaguchi University, 1677–1 Yoshida, Yamaguchi, Japan; Universidade Federal do Rio de Janeiro, BRAZIL

## Abstract

Reactive oxygen species (ROS) are generated spontaneously in all organisms and cause oxidative damage to biomolecules when present in excess. Accumulated oxidative damage accelerates aging; enhanced antioxidant capacity may be a positive factor for longevity. Recently, numerous studies of aging and longevity have been performed using short-lived animals, however, longevity mechanisms remain unknown. Here we show that a termite *Reticulitermes speratus* that is thought to be long-lived eusocial insect than other solitary insects uses large quantities of uric acid as an antioxidant against ROS. We demonstrated that the accumulation of uric acid considerably increases the free radical-scavenging activity and resistance against ultraviolet-induced oxidative stress in laboratory-maintained termites. In addition, we found that externally administered uric acid aided termite survival under highly oxidative conditions. The present data demonstrates that in addition to nutritional and metabolic roles, uric acid is an essential antioxidant for survival and contributes significantly to longevity. Uric acid also plays important roles in primates but causes gout when present in excess in humans. Further longevity studies of long-lived organisms may provide important breakthroughs with human health applications.

## Introduction

Numerous studies of longevity and lifespan extension have been performed using various organisms, including yeast, fruit fly, nematodes, and mice, and various metabolic pathways and molecules have been implicated in life span extension [1]. In particular, growth hormone, insulin, insulin like growth factor-1, and target of rapamycin pathways have been actively investigated, and sirtuin genes have been identified as ‘longevity genes’ that are upregulated in animals whose lives have been prolonged by caloric restriction [2]. Sirtuins are NAD^+^-dependent protein deacetylases that regulate various downstream molecules and are highly conserved from yeast to human [3]. In addition to life span-extension using caloric restriction, long-lived organisms have been generated by genetic engineering [4].

Yeast, fruit fly, nematodes, and mice are ideal models for studying the mechanisms of longevity, with relatively short lifespan and short gestation times. However, novel animal models have recently been made applicable for longevity studies following the development of techniques, such as new generation sequencing analysis. Among these, the naked mole rat *Heterocephalus glaber* and the blind mole rats *Spalax sp*. and *Nannospalax sp*. are subterranean eusocial rodents with extraordinarily long lifespans and resistance to tumorigenesis [5]. Although several studies of these animals are underway, the core mechanisms of longevity remain unidentified. Various eusocial insects are long-lived and reproductive and non-reproductive individuals contribute distinct services to colonies of ants, bees, wasps, and termites. In particular, the lifespans of reproductive and non-reproductive termites differ by 10–100 fold, although the longevity of non-reproductive termites is much longer than that of common solitary insects [6–8]. Although extensions of life span have been of interest for a long time, the mechanisms that contribute to longevity remain unknown.

In this study, we investigated the contribution of oxidative defences to the longevity of termites. Specifically, reactive oxygen species (ROS) are generated spontaneously during normal metabolic activities, and excessive ROS generation causes damage to biomolecules, such as DNA, proteins, and lipids. Accordingly, accumulated oxidative damage has been shown to reduce lifespan in mice, insects, yeast, and nematodes, whereas enhanced antioxidant capacity contributes to longevity [9,10]. In addition, immotile plants have various antioxidants such as ascorbic acid and flavonoids that protect against strong oxidative stress in harsh environments. In the present study, we hypothesised that long-lived termites have strong oxidative defence mechanisms and showed that the common Japanese termite *R*. *speratus* produces large quantities of the strong antioxidant uric acid, which contributes to longevity.

## Materials and methods

### Samples and treatments

Termites (*Reticulitermes speratus*), mantises (*Tenodera aridifolia*) and ants (*Camponotus obscuripes*) were collected in the campus of Yamaguchi University. Fruit flies (*Drosophila melanogaster*) and silkworms (*Bombyx mori*) were a kind gift from Prof. R. Murakami and Prof. J. Kobayashi at Yamaguchi University, respectively. Hornets (*Vespa simillima*) were obtained from a pest exterminator. Insect samples were divided into categories of sex, developmental stage, and caste, and were stored at −80°C until use.

Frozen samples were powdered in liquid nitrogen using a mortar and pestle. After dissolving in sample buffer containing 20 mM Tris-HCl (pH 7.5) and 2% protease inhibition cocktail (Nacalai Tesque, Kyoto, Japan), samples in tubes were homogenized further using a sonicator (TOMY UD-201, Tokyo, Japan) on ice. Samples were then centrifuged at 17,000 × *g* for 10 min at 4°C, and protein concentrations in supernatants were determined using BCA Protein Assay Kits (ThermoFischer scientific, Massachusetts, USA).

Heat- and proteinase- resistance tests were performed by boiling the extracts from *R*. *speratus* soldiers for 10 min or by treating them with 2% proteinase K (Takara, Kusatsu, Japan) and 0.3% sodium dodecyl sulphate (Wako, Tokyo, Japan) followed by warming to 55°C.

Oxidative damage analyses were performed using UV-B irradiation (312 nm, 10.4 kJ/ m^2^) with a UV irradiator (Vilber Lourmat TF-20M, Eberhardzell, Germany). In these experiments, five individual termites per group were prepared on petri dishes and were irradiated equally. Termites remained alive after irradiation.

For termite survival assays, 30 workers or soldiers were maintained on cellulose paper in 35 mm dishes. In allopurinol-feeding experiments, 1 mL of 100 mM allopurinol (Wako, Tokyo, Japan) was dropped on the cellulose paper and was consumed with the paper. Uric acid feeding was administered by dropping 1 mL of 0.6 mM uric acid suspension onto cellulose papers, and 1 mL of 100 mM allopurinol in 0.6 mM uric acid suspension was dropped onto cellulose papers of the cotreatment group. DW was used as a control group. In experiments with paraquat feeding, 0.05 M paraquat, and/or 0.05 M uric acid were dropped onto cellulose paper in the same manner.

### Free radical scavenging activity assay

Antioxidant activities of soluble insect extracts were evaluated using 1, 1- diphenyl-2-picryl hydrazyl (DPPH) radical scavenging assays according to established protocols with modifications [11]. Briefly, frozen insects were powdered in liquid nitrogen and samples were extracted using 500 μL of deionized water. Subsequently, 50 μL samples containing 7.5 μg of protein and 0.02–0.2 mM trolox standards were prepared in 96-well plates, and 100 μL aliquots of 0.15 mM DPPH (Merck Millipore, Massachusetts, USA) solution in EtOH was added to the wells. After incubating for 30 min at 25°C, absorbance of plate wells was measured at 517 nm using a spectrophotometer. Free radical scavenging activities were calculated as trolox equivalents (μM)/protein weight (mg).

### *In vivo* imaging of reactive oxygen species (ROS)

*In vivo* detection of ROS in termite bodies was achieved by injecting the ROS-sensitive reagent dihydrorhodamine 123 (DHR123; ThermoFischer scientific, Massachusetts, USA). Briefly, 25 μM (or 0–40 μM only in supplement figure) DHR123 was prepared and loaded into a glass capillary (Drummond, Pennsylvania, USA). DHR123 solution was then injected into the thorax of CO_2_-anesthetised termites using a FemtoJet microinjector (Eppendorf, Hamburg, Germany). Injected termites were then subjected to UV irradiation for 0, 5, and 10 min (312 nm; 0, 2.6, and 5.2 kJ/m^2^, respectively; Vilber Lourmat TF-20M) through trial- and- error, and fluorescence was observed using a fluorescence microscope (Leica AF 6000 macro modular system, Wetzlar, Germany). Fluorescent intensities of saved images were analysed and quantified using Image J. In this injection method, the injection volume was controlled throughout the series of experiments by using the same glass capillary.

### Thiobarbituric acid reactive substances (TBARS) assay

Oxidative damage was assessed according to lipid peroxidation using the methods reported by Iuchi *et al*. [12] with TBARS assay kits (Cayman chemical, Michigan, USA). Briefly, whole termite worker bodies were UV irradiated for 20 min (312 nm; 10.4 kJ/m^2^) according to a previous method [13] and subsequently homogenised in 200 μL of ice-cold buffer containing 20 mM Tris-HCl (pH 7.5) and 2% protease inhibitor cocktail. Malondialdehyde (MDA) standards and samples were mixed with 50 μL of 10% SDS solution and 1 mL of colour reagent containing 0.53% thiobarbituric acid, 10% acetic acid, and 1.5% sodium hydroxide, and were incubated for 30 min at 100°C. Samples were then incubated on ice for 10 min to stop the reaction and were finally centrifuged at 17,000 × *g* for 10 min at 25°C. Absorbance of the obtained supernatants was determined at 532 nm and TBARS levels were calculated from a MDA standard curve.

### HPLC and LC-MS/MS analysis

358.1 mg of *R*. *speratus* termite soldiers were homogenised by sonication (TOMY UD-201) in 5.0 mL of ice-cold LC/MS grade water (Kanto chemical, Tokyo, Japan). After centrifugation, supernatants were collected and cleared through 0.22 μm filters (Millex^®^GP, Merck Millipore, Massachusetts, USA) for HPLC analyses. Sample extracts were then applied to a Superiorex ODS 5 m (4.6 × 150 mm) column (Shiseido, Tokyo, Japan) and were analysed using a LC-20A system (Shimadzu Scientific Instrument, Tokyo, Japan) under the following conditions: Column temperature, 40°C; injection volume, 10 mL; flow rate, 0.6 mL/min, detection wavelength, 190–320 nm; mode, gradient mode using 0.05% acetic acid (A) and 100% methanol (B) as the mobile phase. In these gradient analyses, the starting solvent was 95% (A) and the concentration of (B) was then increased to 25% over 4.5 min. The concentration of (B) was then maintained at 25% for 1.5 min and was then decreased to 5% over 10 min. Elutes were fractionated and collected every 10 s and antioxidant activities of fractions were measured using DPPH assays.

To identify the termite antioxidant, samples were analysed using a 3200 Q TRAP LC/MS/MS system (AB Sciex Pte. Ltd., Massachusetts, USA) equipped with an ESI interface operated in negative-ion mode. Termite antioxidants were analysed in accordance with the method of Kim *et al*. [14] Analytical conditions were configured as for HPLC analyses, and uric acid (Wako) in 5 mM ammonia solution was used as a standard. LC samples were obtained from *R*. *speratus* soldiers and were further purified using thin-layer chromatography (TLC Silica gel 60 RP-18 F254s, EMD Millipore). Uric acid standard and LC samples were analysed in negative electrospray ionization (ESI) mode and the chemical structure was identified using a multiple reaction monitoring-enhanced product ion (MRM-EPI) scan. The parent ion of uric acid was m/z 167.0 and the major EPI was m/z 124.0.

### Quantification of uric acid contents

Termite uric acid contents were quantified using Uric acid-C kits (Wako, Tokyo, Japan) according to the manufacturer’s instructions. Briefly, 3–5 individuals were desiccated by sonication in 0.5 mL of DW and were then centrifuged at 17,000 × *g* for 10 min at 4°C. Subsequently, 300 μL supernatants were retrieved as sample extracts, and 50 μL aliquots were mixed with working reagent and incubated for 10 min at 37°C. Uric acid contents were determined colorimetrically and were compared with serially diluted standard uric acid solutions.

### Statistical analysis

Statistical tests and significances are specified in every figure legend. Most statistical analyses were performed using R software package (version 3.2.2). Multiple comparisons were performed using unpaired two-tailed *t*-tests followed by *P* value correction using Holm’s method [15]. Data are presented as means ± standard errors of the mean (SEM), and all calculated *P* values are presented in the figure legends. Survival tests were performed using Peto-Peto and Cochran-Mantel-Haenszel log -rank methods in Excel (Microsoft, Washington, USA).

## Results

### Termites have high free radical scavenging activity

**Fig 1:**
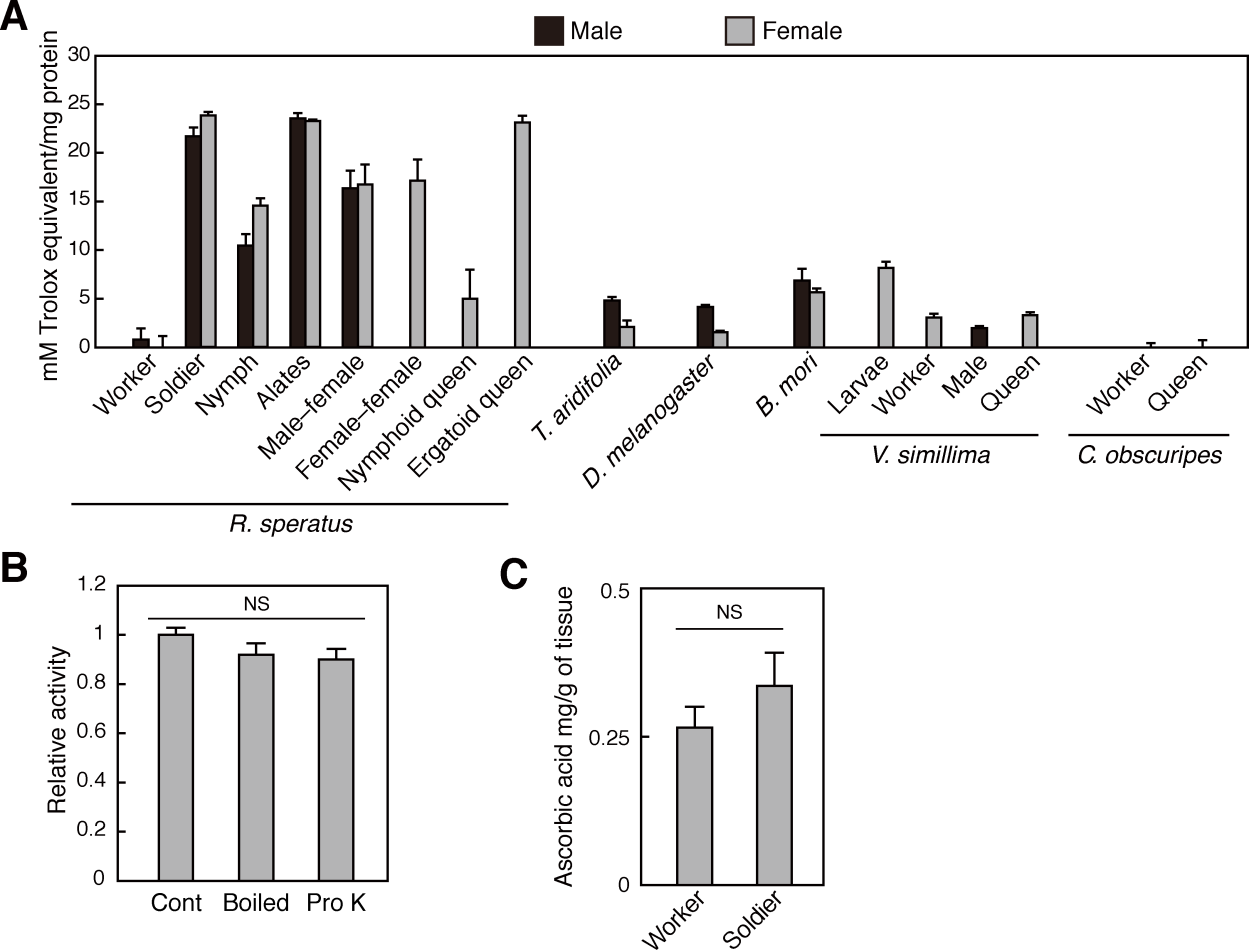
Strong antioxidants in termites *Reticulitermes speratus*. (A) Free radical scavenging activities in various species of insects; soluble extracts from *R*. *speratus*, mantis (*Tenodera aridifolia*), fruit fly (*Drosophila melanogaster*), silkworm (*Bombyx mori*), hornet (*Vespa simillima*) and ant (*Pristomyrmex punctatus*) were used for DPPH radical scavenging assays. Male–female, male–female paired young primary reproductives; Female–female, female–female paired young primary reproductives. Black boxes indicate male and grey boxes indicate female (n = 3–6). (B) The antioxidant activity of termite soldiers after the control, heat (boiled), and proteinase (Pro K) treatments (n = 3–6). (C) The ascorbic acid contents of termite workers and soldiers (n = 3). (D) Data are presented as means ± s.e.m. Statistical significance was assayed using the unpaired *t*-test followed by Holm’s adjustment [no significance (ns)].

Initially, we determined total antioxidant capacities in soluble extracts from whole bodies of various insect species, including eusocial species. DPPH free radical scavenging assays revealed that the Japanese termite *R*. *speratus* has high antioxidant activity compared with other insects (Fig 1A). This high activity was detected in bodies of all termite castes apart from workers. Moreover, the heat- and proteinase-resistant antioxidant in these termites was not ascorbic acid (Fig 1B and 1C and S1 Fig). The antioxidant accumulated in the abdomen but was present at low levels in the guts of soldier bodies, indicating endogenous generation in individuals (S2 Fig).

### Strong antioxidant protects bodies from oxidative stress in termites

**Fig 2:**
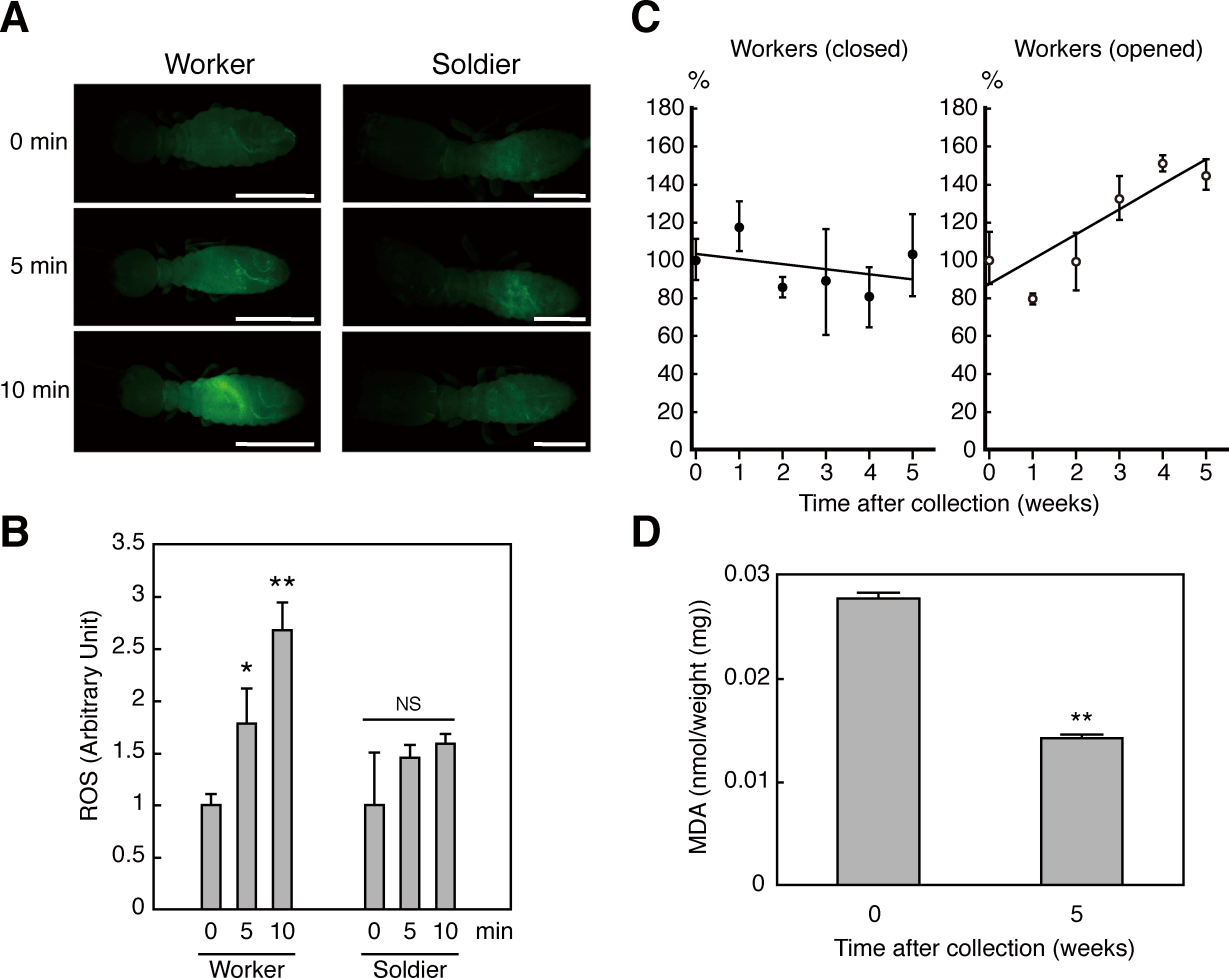
Function of termite antioxidant in the body. (A) Resistance properties of termites against ultraviolet (UV) irradiation; Fluorescence intensities were increased following UV irradiation of termite workers which had lower antioxidant content than termite soldiers; White scales indicate 1 mm. (B) The panel shows fluorescence quantification (n = 6–9). (C) Termite antioxidant activities during captivity in the laboratory; termite workers were maintained in the open (loosely fitted; white) or closed (sealed with tape; black) dish (n = 3). (D) Protective effects of termite antioxidants against lipid peroxidation; lipid peroxidation products were lower in termite workers that had been maintained for 5 weeks than in those that were collected immediately after UV irradiation (n = 3). Data are presented as means ± s.e.m. Statistical significance was assayed using the unpaired *t*-test followed by Holm’s adjustment: *P < 0.05; **P < 0.01.

In subsequent studies, we investigated antioxidant function inside termite bodies using the ROS-sensitive agent dihydrorhodamine123 (DHR123) after microinjection into lateral thoraxes of *R*. *speratus* workers and soldiers (Fig 2A and S3 Fig). After ultraviolet (UV) irradiation producing ROS [16] for 0, 5, and 10 min, fluorescence intensity was measured using a fluorescent microscope. These analyses showed low quantities of the antioxidant in workers and concomitant sensitivity to UV irradiation. In contrast, soldiers containing high amounts of the antioxidant were more resistant to UV irradiation than workers (Fig 2B). These data suggest the possibility that this antioxidant protects termites from oxidative stress generated by UV irradiation. To investigate the importance of this termite antioxidant in oxidative stress defence system, we subjected termites to varying oxidative conditions and measured changes in antioxidant quantities in termite bodies after maintenance under aerobic (opened) and anaerobic (closed) conditions. Free radical scavenging activity of the termite antioxidant was gradually increased in workers that were maintained under aerobic conditions (Fig 2C). In further experiments, we investigated whether the termite antioxidant is protective against oxidative damage to biomolecules, and measured lipid peroxidation products using assays of thiobarbituric acid reactive substances TBARS assays. After the induction of oxidative stress by UV irradiation, the lipid peroxidation marker malondialdehyde (MDA) was present at lower concentrations in workers that had been maintained under laboratory conditions for 5 weeks than in freshly collected individuals (Fig 2D), suggesting that biomolecules in termite bodies are protected by this antioxidant.

### Uric acid is an antioxidant in termites

**Fig 3:**
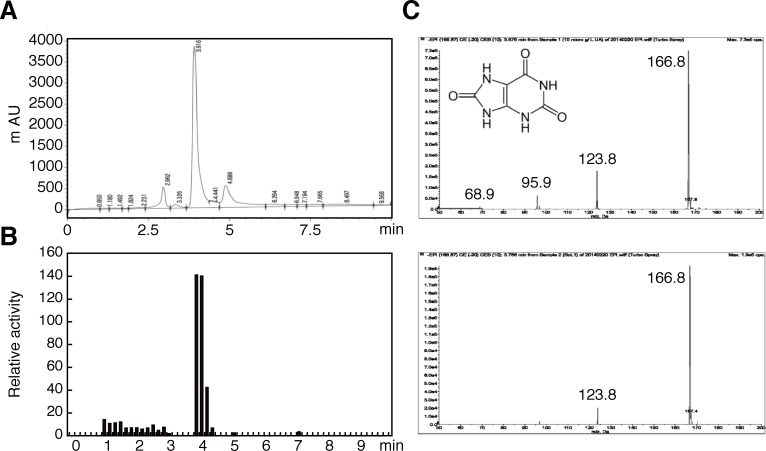
Detection and characterisation of the termite antioxidant. (A) Chromatogram from HPLC analyses and (B) free radical scavenging activities of collected samples fractionated by HPLC; the evident peak of activity was meshed with the highest peak of the HPLC chromatogram. (C) MS-spectra from LC-MS/MS analyses; fragment ion peaks of standard uric acid (upper panel) were completely matched with those of samples from termite soldiers (lower panel).

In a previous report, uric acid gradually accumulated inside termite bodies after maintenance in a laboratory for a long time [17]. Uric acid is also known to exhibit strong free radical-scavenging activity in humans and several insects [18–20].Therefore, we hypothesized that the increased antioxidant might be uric acid in laboratory-maintained workers. To determine the role of uric acid in the observed antioxidant activities of termites, soluble extracts from termite soldiers were fractionated using HPLC (Fig 3A) and free radical scavenging activities of the collected samples were measured. In these experiments, peak free radical scavenging activities (Fig 3B) corresponded with the highest peak for uric acid in HPLC fractions. Subsequently, this peak fraction was applied to LC-MS/MS analyses and the implied antioxidant was compared with a uric acid standard. These analyses indicated that peaks of the fragmented ion from termite samples matched those of the uric acid standard (Fig 3C), indicating that the major antioxidant in termites is uric acid (product ion-monitoring data are shown in S4 Fig). In further experiments, we determined the proportion of total antioxidant activity that can be ascribed to uric acid by treating extracts with the uric acid conversion enzyme uricase (S5A Fig). These experiments showed that antioxidant activities in extracts diminished with decrease in uric acid concentrations (S5B Fig), suggesting that uric acid is a pivotal antioxidant in termite bodies.

### Uric acid enhances termite worker survival in laboratory assays

Although termite workers have comparatively low levels of uric acid immediately after collection from the field, laboratory-maintained workers accumulated uric acid [17]. Consistent with these previous reports, the body colour of *R*. *speratus* workers gradually changed to white under laboratory conditions (S6A Fig). Consequentially, we compared UV resistance of three groups of workers after maintenance under laboratory conditions at various times. In these experiments, uric acid levels and antioxidant activities increased with time in laboratory-maintained workers (S6B and S6C Fig), and ROS levels decreased inside the body (S6D Fig). Because laboratory conditions (open air) may be more aerobic than the insides of termite colonies (confined spaces), the present increases in antioxidant activity due to uric acid accumulation are likely an adaptation to oxidative stress.

**Fig 4:**
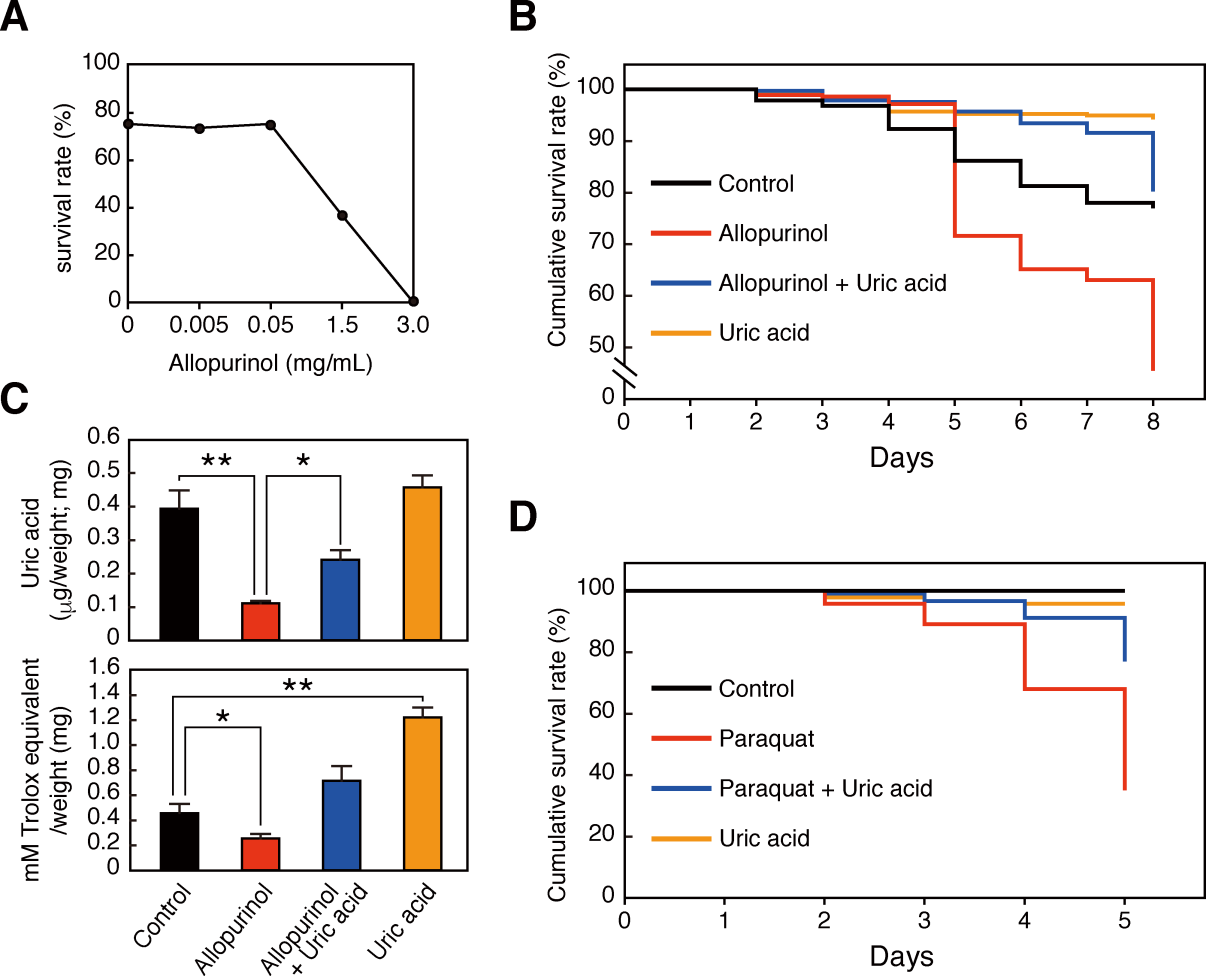
Uric acid contributes to the survival of termite worker under laboratory conditions. (A) Survival rates of termite workers following allopurinol administration for 10 days. (B) Effects of allopurinol and/or uric acid administration on termite survival rates; Feeding of termite workers with the uric acid synthesis inhibitor allopurinol resulted in decreased survival rates (red line). Treatment with allopurinol and uric acid dramatically arrested allopurinol-mediated decreases in survival rates (blue line; n = 270). All treatment groups differed significantly *P* < 0.001, except that no differences were identified between control (black line) and allopurinol + uric acid groups. (C) Uric acid contents (upper panel) and antioxidant activities (lower panel) of treatment groups at 8 days (n = 3). All error bars represent means ± SEM. Statistical significance was assayed using the unpaired t-test followed by Holm's adjustment: *P < 0.05, **P < 0.01. (D) Feeding of termite workers with paraquat resulted in decreased survival rates (red line); however, co-treatment with uric acid dramatically rescued paraquat-induced reductions in survival rates (blue line; n = 90). All comparisons revealed significant differences (*P* < 0.001), except for that between the control group and the uric acid group. *P* values were obtained using log-rank tests (Peto–Peto test and Cochran–Mantel–Haenszel test) and Peto–Prentice–Wilcoxon tests.

Finally, we investigated whether termite uric acid is essential for survival by monitoring survival of termite workers in the presence of the uric acid synthesis inhibitor allopurinol. Allopurinol dose-dependently decreased survival, suggesting that uric acid depletion from worker bodies leads to death (Fig 4A). Externally administered antioxidants, including vitamin C, E, and some flavonoids reportedly improved longevity in various animal models [21]. Thus, in further experiments, we examined the effect of added uric acid and observed positive effects on termite longevity. Specifically, orally ingested uric acid rescued survival rates following allopurinol intake (Fig 4B). Moreover, decreased uric acid contents and free radical scavenging activities of allopurinol-treated termite bodies were recovered by uric acid intake (Fig 4C).

Given that uric acid has been considered an important nitrogen source for many insects, including termites [22], uric acid depletion by allopurinol may lead to death irrespective of its antioxidant activities. Thus, to investigate whether uric acid improves termite longevity under highly oxidative conditions, and to distinguish between antioxidant and nutritional roles, termite workers were fed uric acid concurrently with the pro-oxidant paraquat. The survival curves in Fig 4D indicate that uric acid intake rescues termite survival rates in the presence of paraquat, suggesting that uric acid contributes to termite longevity essentially as an antioxidant.

## Discussion

Herein, we have presented evidence that in addition to roles as a nutritional source of nitrogen, large quantities of uric acid in some termite castes play antioxidant roles. Furthermore, we demonstrate that uric acid positively participates in termite longevity and stress responses. Uric acid is the main end product of nitrogen metabolism in insects, reptiles and birds, whereas the main end product in mammals is urea. Uric acid functions as a major plasma antioxidant, and is the end product of purine metabolism in most primates including humans. Several insect species use uric acid as a protective compound. Accordingly, body colour of the silkworm *Bombyx mori* is reportedly influenced by epithelial uric acid, which protects against UV radiation from the sun. Several uric acid-deficient silkworm mutants have been generated in previous studies and these mutants have translucent skin owing to a deficiency in xanthine dehydrogenase, which synthesises uric acid [23]. Uric acid contents in silkworms were also reduced after feeding with the specific xanthine dehydrogenase inhibitor allopurinol, and uric acid-depleted silkworms were hypersusceptible to UV irradiation [19]. Moreover, in experiments with the fruit fly *Drosophila melanogaster*, the uric acid-deficient mutant *rosy* was susceptible to high temperatures and oxidative stress [20]. These data suggest that uric acid plays important antioxidant roles in addition to those as a metabolic product. Thus, to investigate the function of uric acid as a defence mechanism, we assessed the internal redox status of termite bodies using a ROS-sensitive fluorescent reagent, and visualised *in vivo* oxidative stress in whole insect bodies.

UV irradiation is an artificial source of oxidative stress for termite species, because termite workers and soldiers are rarely exposed to sunlight. Thus, to model physiologically relevant oxidative stress, we reduced uric acid in termites using allopurinol and examined survival. Allopurinol intake markedly reduced termite survival rates in a dose-dependent manner (Fig 4A), reflecting the importance of uric acid. Because allopurinol has a purine skeleton, it also acts on enzymes of nucleic acid metabolism, likely leading to toxicity and decreased longevity irrespective of uric acid depletion. Thus, to distinguish between these effects we supplemented allopurinol-treated termite workers with uric acid. Under these conditions, uric acid administration rescued survival, indicating that even exogenous uric acid can compensate for deficiencies in uric acid production, and that endogenous uric acid is positively associated with survival. To determine whether uric acid functions as an antioxidant inside termite bodies, we performed experiments with the toxic pro-oxidant paraquat and observed decreased termite survival that again could be arrested by intake of uric acid. These observations indicate that uric acid protects against paraquat toxicity, possibly reducing oxidative stress by scavenging paraquat-mediated ROS. Furthermore, absolute uric acid intake was associated with increased survival rates, indicating survival advantages of increased uric acid contents. Previous studies suggest that termite uric acid is degraded by intestinal anaerobic bacteria and is reused as a source of carbon, nitrogen, and energy [22,24]. As a potential mechanism for uric acid accumulation, termites that are maintained outside of the subterranean colony may experience increased oxidative stress under more aerobic conditions potentially leading to decreases in intestinal anaerobic bacteria and slowed uric acid degradation. This hypothetical mechanism could effectively upregulate antioxidant activities in termites that are exposed to aerobic conditions.

Taken together, the present data indicate that uric acid contributes to termite longevity. Specifically, whole termite castes had large quantities of uric acid. However, female nymphoid neotenics (secondary queens) and primary kings had lower uric acid contents, suggesting the presence of other mechanisms of longevity, and warranting further analyses of antioxidant activities and enzymes and longevity in reproductive termites [13]. It remains unknown how and where uric acid functions in termite bodies. In addition, high accumulation of poorly soluble uric acid may lead to crystallisation, although the present data suggest that termites can regulate the solubility of uric acid. Investigations of the associated mechanisms of uric acid solubility may lead to interventions that dissolve crystallised uric acid and improve conditions for humans with gout.

## Supporting information

S1 Fig Free radical scavenging activities of heated samples from various insect species. Soluble extracts from termite (*Reticulitermes speratus*), mantis (*Tenodera aridifolia*), fruit fly (*Drosophila melanogaster*), silkworm (*Bombyx mori*), yellow hornet (*Vespa simillima*), and ant (*Camponotus obscuripes*) were heated to boiling, and were then analysed in DPPH radical scavenging assays. Termites generally had higher antioxidant activities. Black boxes indicate male, and grey boxes indicate female (n = 3–6).
(TIF)

S2 Fig Antioxidants are located mainly in abdominal parts except for the gut. Antioxidant activity of head, thorax, abdomen, and gut of termite soldiers (n = 3). Black scales indicate 1 mm. Data are presented as means ± SEM. Statistical significance was assayed using the unpaired *t*-test followed by Holm's adjustment: **P < 0.01.
(TIF)

S3 Fig *In vivo* ROS detection by DHR123. Fluorescence images of termite workers injected with serial concentrations of dihydrorhodamine 123 and subjected to UV irradiation. The lower panel shows image quantifications (n = 6–9).
(TIF)

S4 Fig MRM analysis between uric acid and termite soldier. MRM analysis of the uric acid standard (upper panel) and soluble extracts of termite soldiers (lower panel). The retardation time of MRM peak was matched with that of the uric acid standard.
(TIF)

S5 Fig Free radical scavenging activities depends on uric acid contents in termites. (A) Uric acid contents in soluble extracts from termite soldiers; free radical scavenging activities of soldier extracts after treatment with uricase (n = 3). (B) Uric acid contents were significantly decreased in uricase-treated samples (n = 3). Data are presented as means ± SEM. Statistical significance was assayed using the unpaired *t*-test: **P < 0.01.
(TIF)

S6 Fig Function of uric acid in termite bodies. (A) Body colours of termite workers changed to white during captivity in the laboratory. (B) Laboratory-maintained termites accumulated uric acid in their bodies during captivity. (C) Increase in antioxidant activities in laboratory-maintained termites. (D) ROS generated by UV irradiation were suppressed in the bodies of uric acid-accumulated termites. Data are represented as means ± SEM. Statistical significance was assayed using the unpaired *t*-test followed by Holm's adjustment: *P < 0.05, **P < 0.01.
(TIF)
