# Supplementary figures and images for: Uric acid, an important antioxidant contributing to survival in termites
Source: PLoS One. 2017 Jun 13;12(6):e0179426. doi: 10.1371/journal.pone.0179426 (PMC5469489; doi:10.1371/journal.pone.0179426)

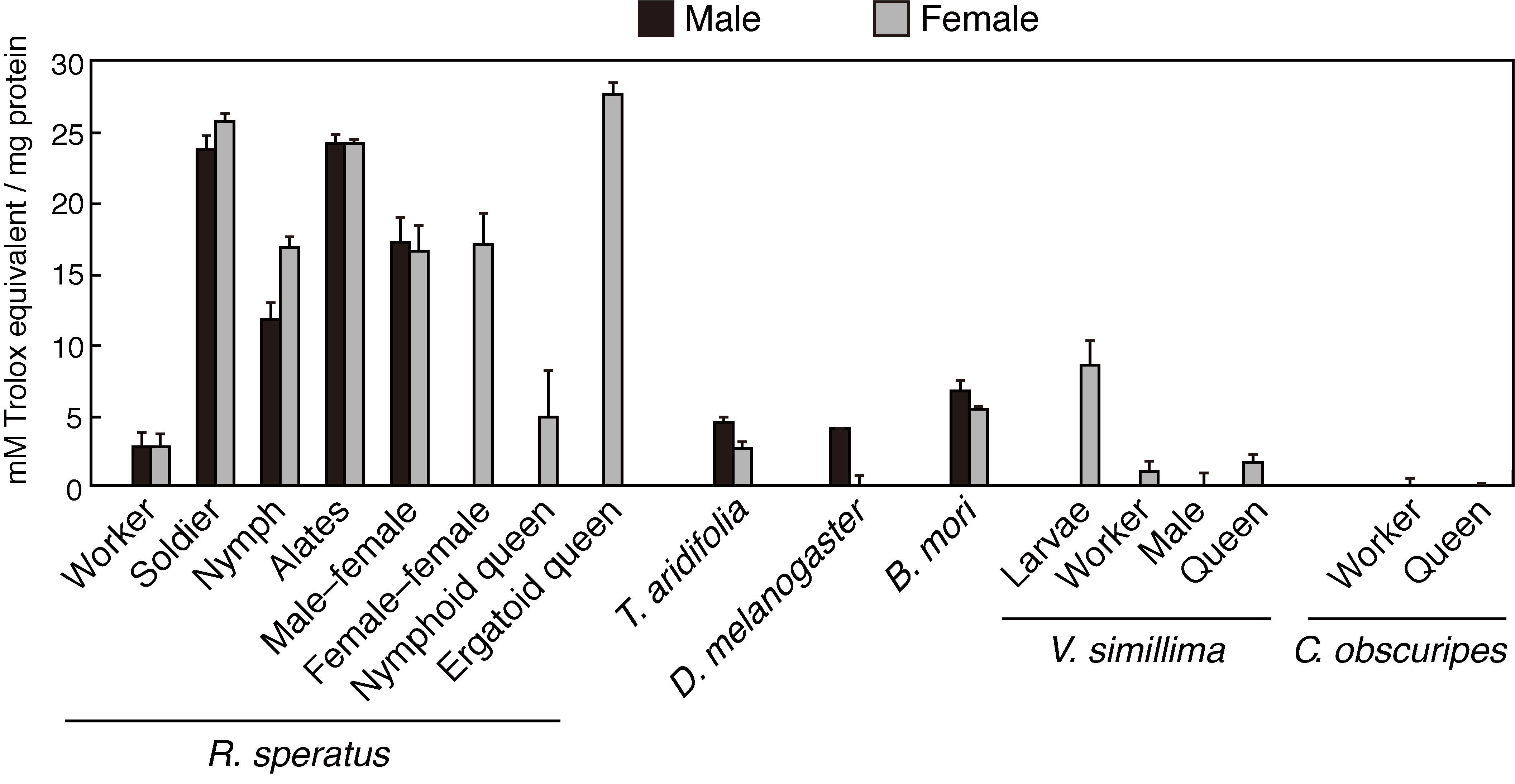

Supplement: S1 Fig — Soluble extracts from termite (Reticulitermes speratus), mantis (Tenodera aridifolia), fruit fly (Drosophila melanogaster), silkworm (Bombyx mori), yellow hornet (Vespa simillima), and ant (Camponotus obscuripes) were heated to boiling, and were then analysed in DPPH radical scavenging assays. Termites generally had higher antioxidant activities. Black boxes indicate male, and grey boxes indicate female (n = 3–6). (TIF) [file pone.0179426.s001.tif]

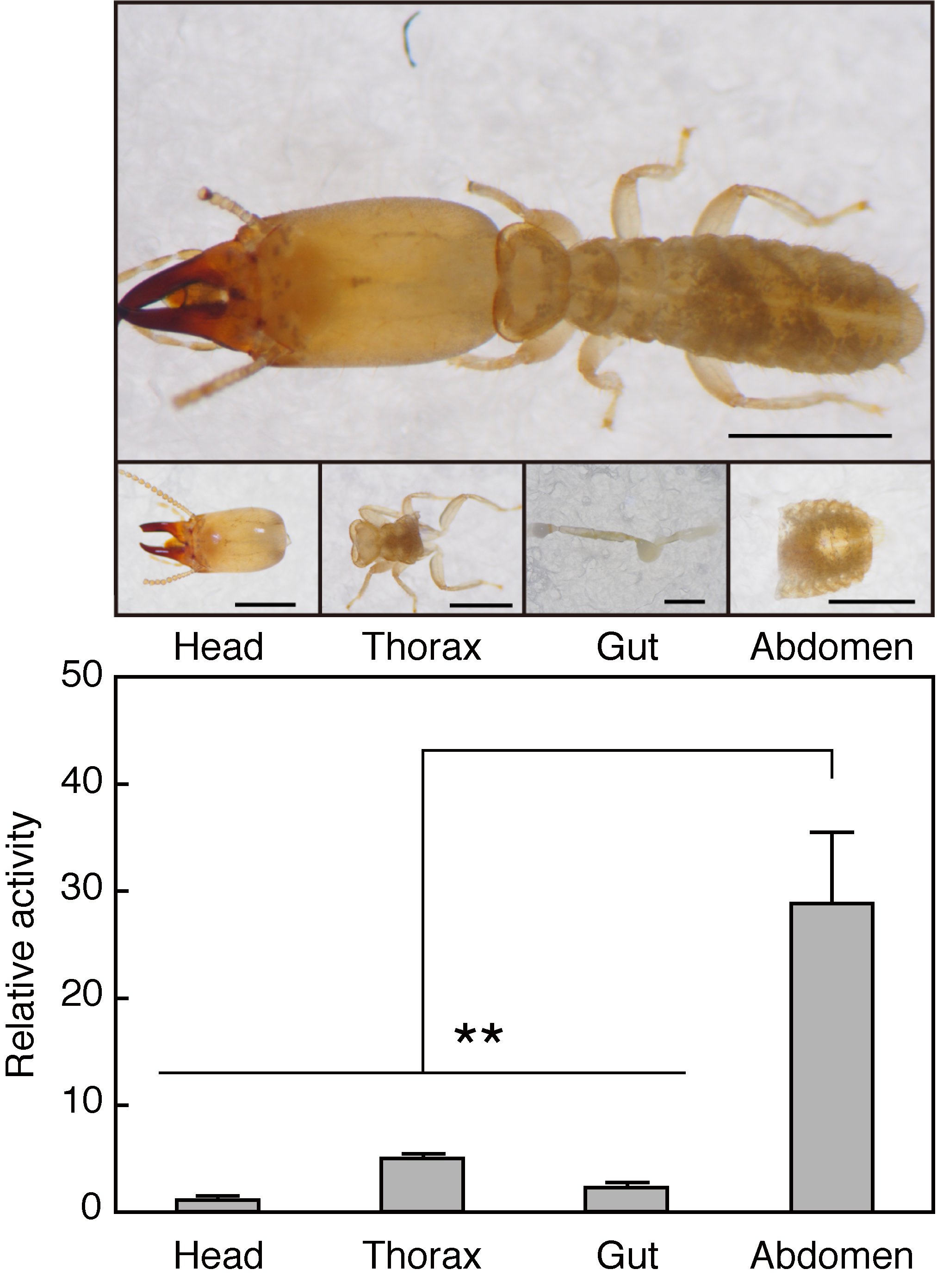

Supplement: S2 Fig — Antioxidant activity of head, thorax, abdomen, and gut of termite soldiers (n = 3). Black scales indicate 1 mm. Data are presented as means ± SEM. Statistical significance was assayed using the unpaired t-test followed by Holm's adjustment: **P < 0.01. (TIF) [file pone.0179426.s002.tif]

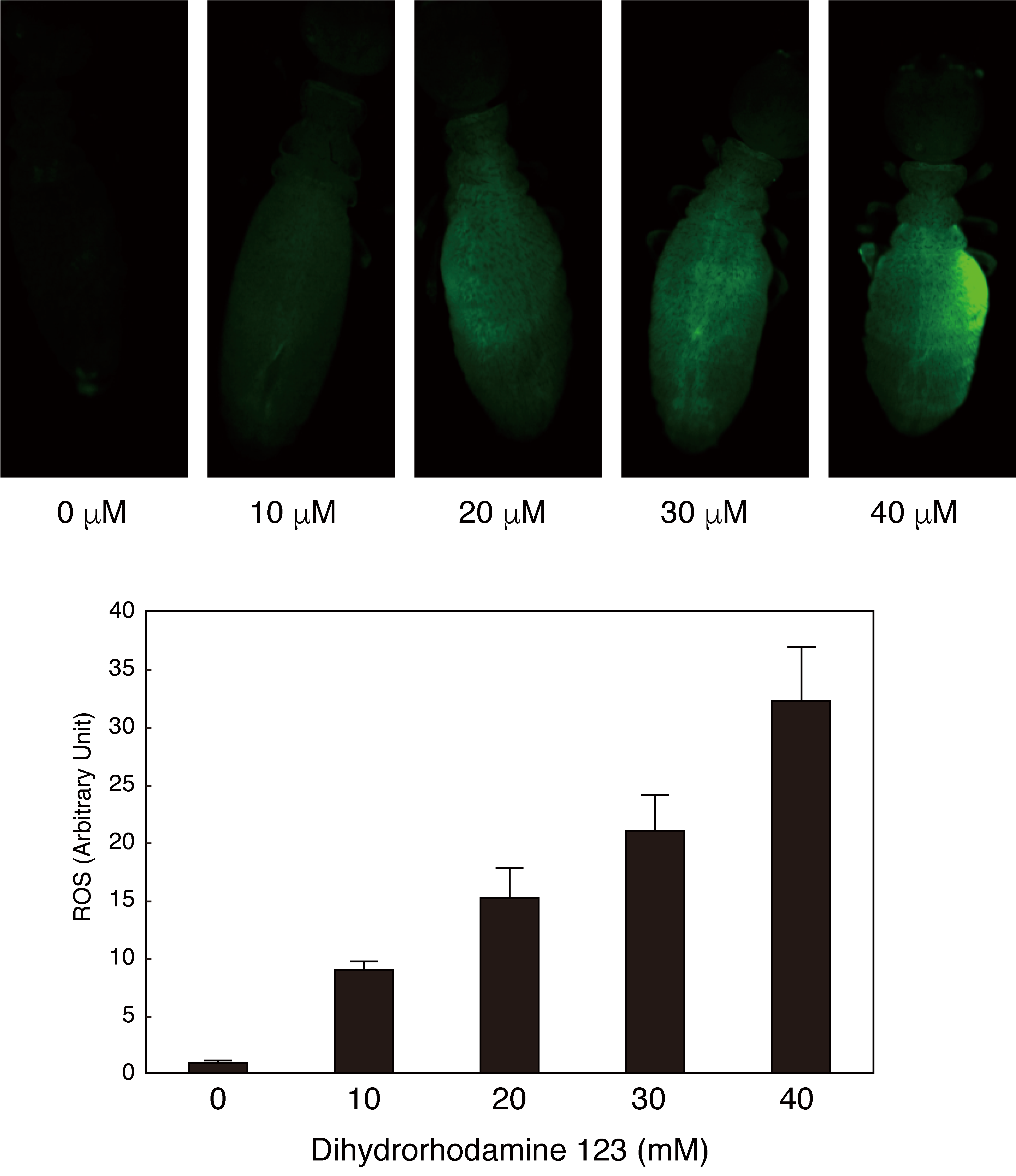

Supplement: S3 Fig — Fluorescence images of termite workers injected with serial concentrations of dihydrorhodamine 123 and subjected to UV irradiation. The lower panel shows image quantifications (n = 6–9). (TIF) [file pone.0179426.s003.tif]

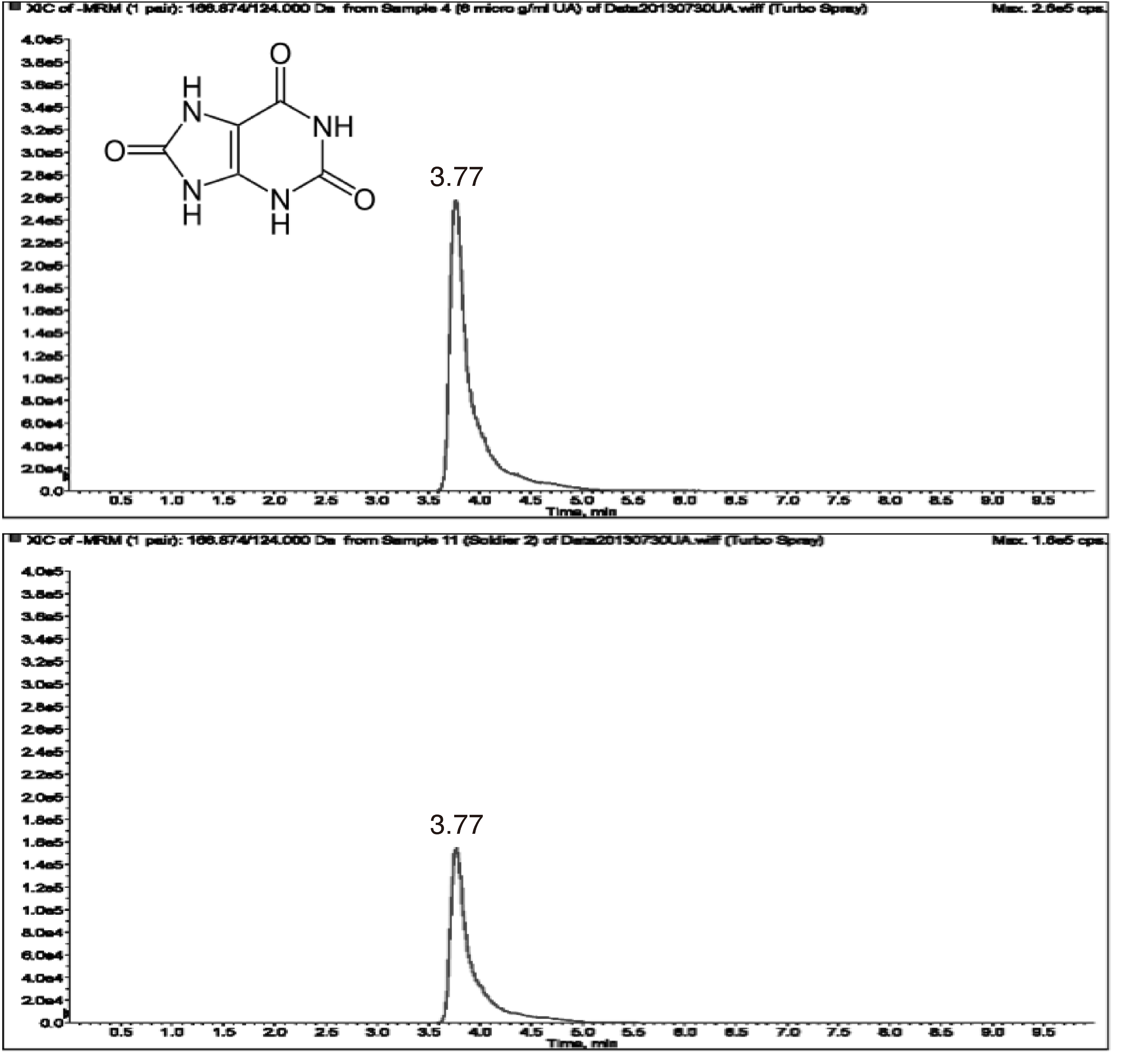

Supplement: S4 Fig — MRM analysis of the uric acid standard (upper panel) and soluble extracts of termite soldiers (lower panel). The retardation time of MRM peak was matched with that of the uric acid standard. (TIF) [file pone.0179426.s004.tif]

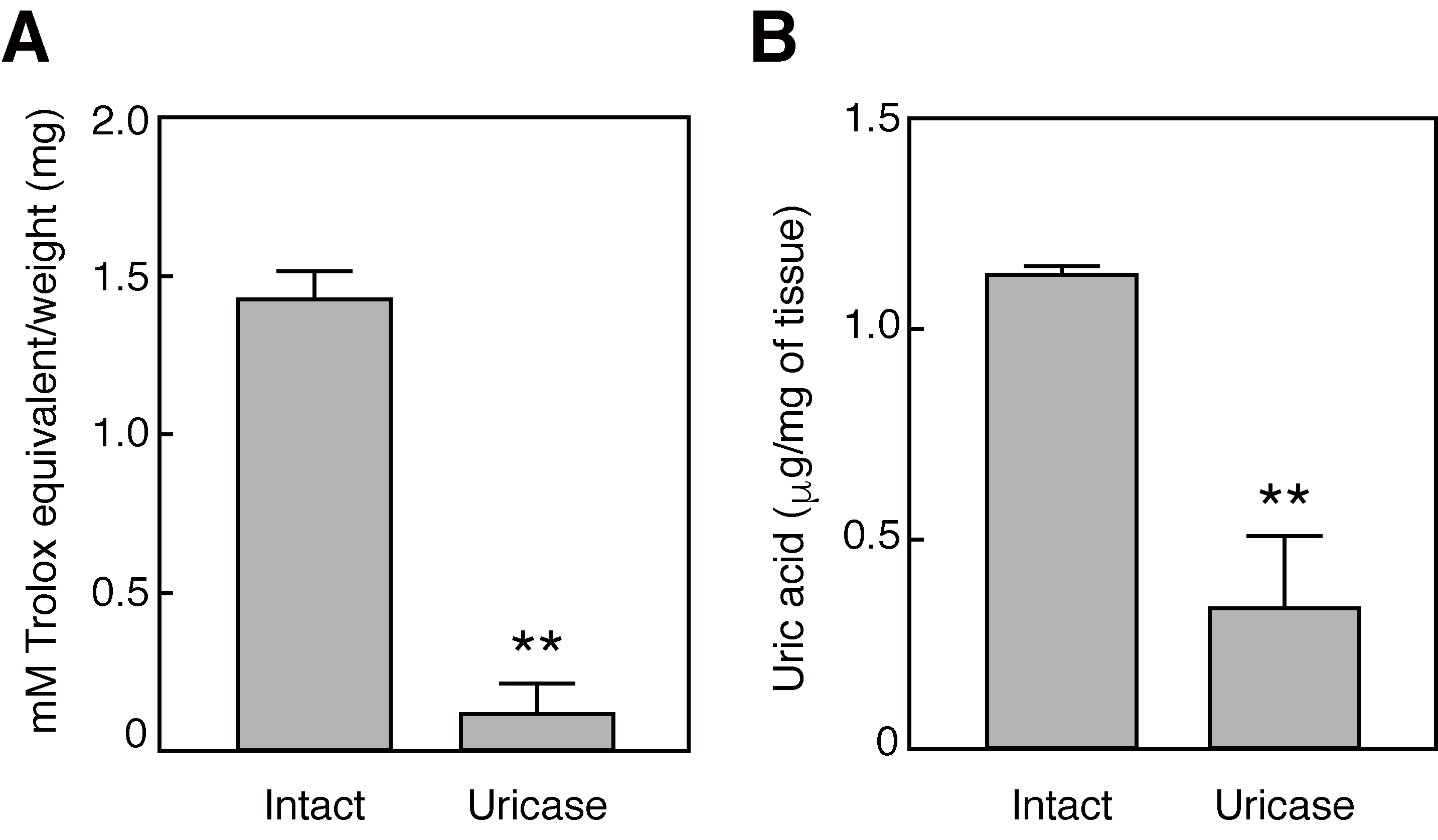

Supplement: S5 Fig — (A) Uric acid contents in soluble extracts from termite soldiers; free radical scavenging activities of soldier extracts after treatment with uricase (n = 3). (B) Uric acid contents were significantly decreased in uricase-treated samples (n = 3). Data are presented as means ± SEM. Statistical significance was assayed using the unpaired t-test: **P < 0.01. (TIF) [file pone.0179426.s005.tif]

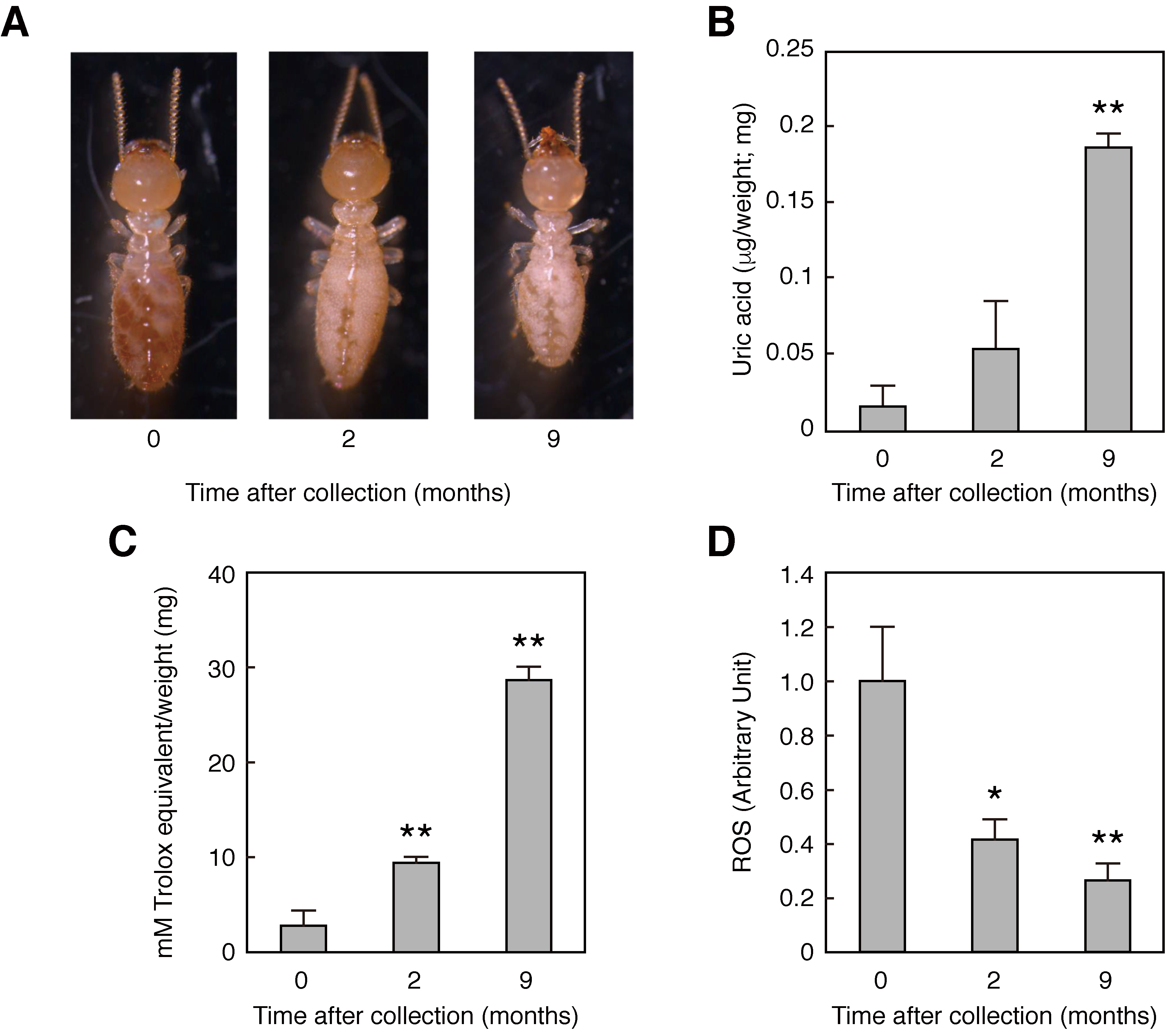

Supplement: S6 Fig — (A) Body colours of termite workers changed to white during captivity in the laboratory. (B) Laboratory-maintained termites accumulated uric acid in their bodies during captivity. (C) Increase in antioxidant activities in laboratory-maintained termites. (D) ROS generated by UV irradiation were suppressed in the bodies of uric acid-accumulated termites. Data are represented as means ± SEM. Statistical significance was assayed using the unpaired t-test followed by Holm's adjustment: *P < 0.05, **P < 0.01. (TIF) [file pone.0179426.s006.tif]
